# Brain stimulation reveals distinct motives underlying reciprocal punishment and reward

**DOI:** 10.1098/rspb.2022.1590

**Published:** 2022-11-09

**Authors:** Leticia Micheli, Marcello Negrini, Teresa Schuhmann, Arno Riedl

**Affiliations:** ^1^ Institute of Psychology, Würzburg University, Würzburg, Germany; ^2^ Department of Marketing and Supply Chain Management & Maastricht University – Center of Neuroeconomics, Maastricht University, Maastricht, The Netherlands; ^3^ Department of Cognitive Neuroscience, Maastricht University, Maastricht, The Netherlands; ^4^ Department of Microeconomics and Public Economics & Maastricht University – Center of Neuroeconomics, Maastricht University, Maastricht, The Netherlands; ^5^ Open Evidence Research, Milan, Italy; ^6^ Paris School of Economics, CNRS, Paris, France

**Keywords:** reciprocal fairness, punishment, reward, DLPFC, mPFC, brain stimulation

## Abstract

Reciprocal fairness, in the form of punishment and reward, is at the core of human societal order. Its underlying neural mechanisms are, however, not fully understood. We systemize suggestive evidence regarding the involvement of the right dorsolateral prefrontal cortex (rDLPFC) and medial prefrontal cortex (mPFC) in reciprocal fairness in three cognitive mechanisms (*cognitive control*, *domain-general* and *self-reference*). We test them and provide novel insights in a comprehensive behavioural experiment with non-invasive brain stimulation where participants can punish greedy actions and reward generous actions. Brain stimulation of either brain area decreases reward and punishment when reciprocation is costly but unexpectedly increases reward when it is non-costly. None of the hypothesized mechanisms fully accounts for the observed behaviour, and the asymmetric involvement of the investigated brain areas in punishment and reward suggests that different psychological mechanisms are underlying punishing selfishness and rewarding generosity. We propose that, for reciprocal punishment, the rDLPFC and the mPFC process self-relevant information, in terms of both personal cost and personal involvement; for reciprocal reward, these brain regions are involved in controlling selfish and pure reciprocity motives, while simultaneously promoting the enforcement of fairness norms. These insights bear importance for endeavours to build biologically plausible models of human behaviour.

## 1. Introduction

Reciprocal fairness is key in organizing human interactions across different cultures [1–3] and permeates our lives from daily exchanges to diplomacy relations. It refers to responding to unkind behaviour with punishment and to kind actions with reward and is essential for discouraging defection and promoting cooperation in societies [4,5]. The importance of reciprocal fairness in human society is also reflected in individuals' willingness to punish and reward not only when they are directly affected by other's actions (second-party reciprocal fairness) [6–9] but also when they are unaffected observers (third-party reciprocal fairness) [10–12], even if such actions are costly [1,4,6–12].

The omnipresence of reciprocal fairness suggests an evolutionarily rooted biological foundation [13,14]. Accordingly, it has been proposed that the prefrontal cortex of the human brain is implicated in the implementation of reciprocal fairness. Specifically, evidence suggests that the right dorsolateral prefrontal cortex (rDLPFC) and medial prefrontal cortex (mPFC) are crucial in the execution of reciprocal punishment [15–22]. However, evidence regarding neural processes underlying reciprocal reward is scarce. Existing studies analysing brain processes related to reciprocal fairness have used varying study designs which make it difficult to draw clear conclusions. These studies investigated either solely second-party reciprocal fairness [15,23] or third-party reciprocal fairness [19,20], or varied two factors simultaneously, like second-party versus third-party interactions together with the cost of reciprocation [21,22]. Moreover, in the reciprocal fairness literature, very little attention has been paid to the neural mechanisms of reward. Consequently, it is still unclear what roles the rDLPFC and the mPFC play in regulating reciprocal reward and whether these are similar to the roles they play in reciprocal punishment. We provide an encompassing study design comprising punishment and reward that varies one factor at a time and allows us to draw comprehensive conclusions about the cognitive mechanisms associated with reciprocal punishment and reward.

## 2. Three proposed cognitive mechanisms involved in reciprocal fairness: *cognitive control*, *domain-general* and *self-reference*

A prominent role of the rDLPFC refers to its involvement in *cognitive control* processing. In the context of reciprocal fairness, cognitive control has been operationalized as the capacity to facilitate the enforcement of a fairness goal, thereby overcoming the impulse to behave selfishly and maximize one's own immediate material gain [15]. In support of this interpretation, there is evidence that brain stimulation over the rDLPFC reduces second-party costly punishment [15] as well as costly reciprocation of trust [23]. Consistent with that, it has also been shown that this region is activated when individuals punish non-cooperative behaviour [24], interact with non-cooperative partners [25] and reject unfair allocations in the ultimatum game [26]. Although the cognitive control mechanism has only been tested in second-party interactions, presumably it is also important in third-party contexts, when enforcing the fairness goal implies material costs.

A different stream of literature suggests that reciprocal fairness is facilitated by *domain-general* cognitive processes for action selection, which have adaptively evolved also to the context of social interactions. Specifically, it has been proposed that in face of third-party norm violations, domain-general cognitive processes generate evaluations of harm and blame. Such evaluations are used to form an intuition of deserved punishment of norm transgressors, which is then integrated by the rDLPFC with the available punishment options in order to select appropriate punishment [27]. Consistent with this interpretation, functional imaging evidence showed that the rDLPFC is involved when disinterested third-party participants respond to individuals who are responsible for harming others and make non-costly punishment decisions in response to such transgressions [19]. A follow-up study demonstrated that transcranial magnetic stimulation (TMS) over the rDLPFC significantly reduced non-costly punishment for wrongful acts in a third-party context by altering the weight attributed to information about harm and blame [20]. Although only considered in a third-party context, this proposed mechanism suggests a general role of the rDLPFC in integrating intuitions of reciprocal responses and selecting a reasonable retributive action. Such action selection of appropriate punitive responses by the rDLPFC should be independent of selfish motives and cost-benefit trade-offs, which differentiates it from the cognitive control hypothesis. Thus, the domain-general mechanism should apply equally in second- and third-party interactions [27].

Regarding the role of the mPFC in reciprocal fairness, it has been proposed that it is implicated in a *self-referential* mechanism. Specifically, in the context of rejections of unfair offers in an ultimatum game (reciprocal punishment), functional imaging evidence shows that the mPFC is significantly more engaged in second- than in third-party interactions, suggesting that it is specially recruited when punishing unfairness that is directed towards oneself [21]. Further evidence supporting this interpretation comes from non-invasive brain stimulation showing that cathodal stimulation over this region decreased punishment only in second-party contexts [22]. This engagement of the mPFC in reciprocal fairness is consistent with neuroscientific evidence which has repeatedly implicated cortical midline structures—including the mPFC—in processing self-referential stimuli, for example during self-reflection [28,29] and evaluative judgements that depend on one's internalized values [30] (see also [31,32]). Hence, this mechanism may be thought of as responding to the salience of any personally relevant outcome.

### (a) Study set-up and hypotheses

We systemized these previous findings on the role of the rDLPFC and the mPFC in reciprocal fairness in a coherent set of preregistered hypotheses (https://osf.io/pg7yc) to test the discussed cognitive mechanisms in a study design which varies only one factor at a time: costs of reciprocation and personal involvement, respectively. Next, we present our study design followed by a description of the tested hypotheses.

We designed a comprehensive behavioural experiment combined with TMS to dissect the involvement of the rDLPFC and the mPFC in reciprocal punishment and reward in second-party and third-party interactions, when reciprocation is costly and when it is non-costly. In a within-subjects design, participants (*n* = 33) took part both as second-party (Receiver) and third-party (Observer), where they could punish or reward a Proposer after having learned how the latter allocated a monetary amount between themselves and the Receiver. Proposers could choose between five bundles of allocations: Very Greedy, Greedy, Equal, Generous and Very Generous. For the Receiver and Observer, punishment or reward of the Proposer was costly or non-costly.

**Figure 1.**
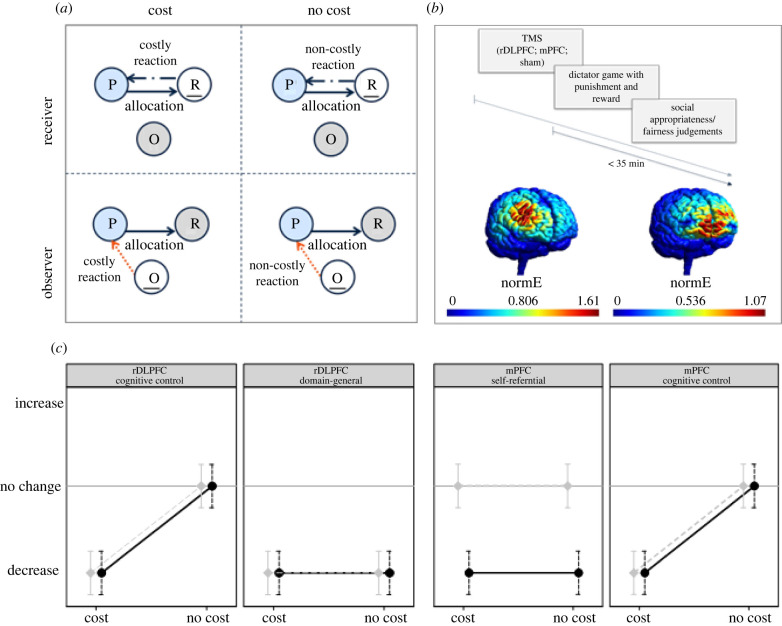
Experimental design. (*a*) Four experimental conditions varying personal involvement and cost of reciprocal fairness. Participants (white circles) are in the role of, respectively, Receiver (R) and Observer (O) (within-subject) and can decide to punish or reward the Proposer (P). Each role has an initial endowment of experimental currency units (ECUs): Proposer, 240 ECU; Receiver, 0 ECU; Observer, 200 ECU. Each ECU invested in punishment/reward reduces/increases the payoff of the Proposer by 5 ECU; *Cost:* payoff of Receiver/Observer decreases by 1 ECU for each ECU invested in punishment/reward; *No cost:* payoff of Receiver/Observer is unaffected. Proposers' allocations were Very Greedy (Proposer: 200, Receiver: 40), Greedy (160, 80), Equal (120, 120), Generous (80, 160) and Very Generous (40, 200); chosen allocations were randomly jittered by ±3 ECU. (*b*) Timeline and SimNIBS TMS simulation of brain areas reached by TMS using the double-cone coil. *Left*: rDLPFC (stimulation site: electrode F4, International 10–20 EEG system); *Right:* mPFC (stimulation between Fp1 and Fp2). Colours show the strength of the normalized TMS electric field (normE) in volts per metre and warmer colours indicate a stronger electric field. (*c*) Graphical illustration of the hypotheses. Predicted effects of TMS over the respective brain area are relative to sham. Black: Receiver; grey: Observer (Online version in colour.)

Each participant attended three different sessions in which TMS was applied to either the rDLPFC or the mPFC or in which sham stimulation was used (counterbalanced order). In each session, participants took part in 140 trials repeatedly facing allocations from all five possible allocation bundles from different Proposers. Each participant made decisions in four different conditions with 35 trials per condition: as a Receiver with costly reciprocation (Rec_C) and non-costly reciprocation (Rec_NC), and as an Observer with costly reciprocation (Obs_C) and non-costly reciprocation (Obs_NC) (figure 1*a*). Thereafter, participants were presented once more with all five possible allocation bundles per condition and asked to rate them in terms of fairness on a 5-point Likert-scale and to rank them according to their social appropriateness in an incentivized task [33]. Figure 1*b* shows the trial timeline.

The proposed cognitive mechanisms generate testable predictions (see figure 1*c* for a graphical representation of the predicted effects; all predictions are formulated relative to the sham condition). In our hypotheses, we assume that TMS over the rDLPFC and mPFC would lead to downregulation of these regions. This is based on previous literature showing that repetitive TMS over the motor cortex has an inhibitory effect as shown by decreased amplitudes of motor evoked potentials [34,35]. We also note that even though the cognitive mechanisms supposedly involved in reciprocal fairness have been proposed in the context of reciprocal punishment, we expect that similar mechanisms will govern reciprocal reward. This is an exploratory assumption, given the lack of studies investigating the neural mechanisms of reciprocal reward and how they compare to those of reciprocal punishment. This assumption appears reasonable given that previous studies have implicated the rDLPFC and mPFC in reciprocation of trust [23] and help [36] and that cognitive control seems to be important also to guarantee costly reciprocation of trust to build a good reputation [23].

First, if the rDLPFC is crucial for controlling immediate material selfishness in reciprocal fairness (*cognitive control* mechanism), TMS over this area should decrease, respectively, punishment of greedy and reward of generous allocations, both in second-party and third-party interactions, when it is costly doing so (Rec_C and Obs_C). Such a decrease is not expected when reciprocation is non-costly (Rec_NC and Obs_NC), because material gain or loss is not at stake there.

Second, if the rDLPFC has a domain-general role facilitating the integration of intuitions about reciprocal fairness and the selection of an appropriate action among a range of response options regardless of cost-benefit calculations (*domain-general* mechanism), TMS over this area should hamper the integration of the information necessary to determine punishment and reward severity. In particular, information about the generosity or greediness of an allocation will be less well integrated, which should weaken punishment and reward in all conditions, regardless of respectively costs of reciprocation and who is the target of the greedy or generous behaviour.

Third, if the mPFC is associated with a *self-referential* mechanism in reciprocal fairness which enhances the salience of personally relevant outcomes irrespective of whether the offers are greedy or generous, TMS over this area should result in a decrease of punishment and reward in second-party interactions, irrespective of the cost of punishment (Rec_C and Rec_NC), but not in third-party interactions. Fourth, previous studies varied second-party and third-party interactions together with costs of punishment [21,22]. Consequently, the involvement of the mPFC in a *cognitive control* mechanism cannot be excluded [22]. If this is the case, TMS over the mPFC should lead to reduced punishment and reward in conditions where reciprocation is costly, regardless of personal involvement (Rec_C and Obs_C).

Finally, previous studies have shown that brain stimulation over the rDLPFC affects reciprocal punishment without altering the perceived fairness of allocations [15,18]. This suggests that the dealing out of punishment and the perception of fairness are guided by distinct cognitive processes and brain regions, with no prefrontal cortex involvement in the latter. Accordingly, we hypothesize that TMS over either brain area does not affect fairness judgements. There exists no evidence regarding the causal effects of brain stimulation of prefrontal brain areas on the social appropriateness of allocations. We assume that similar cognitive processes as in the perception of fairness are at play and consequently expect that TMS over the brain areas of interest does not affect social appropriateness scores.

## 3. Materials and methods

### (a) Experimental procedures

Thirty-three participants (24 women, mean age = 21.9 years, min = 18, max = 33, s.d. = 3.4, 29 right-handed) attended three different sessions, in which they received either TMS over the rDLPFC, or TMS over the mPFC, or sham stimulation. The order of stimulation was counterbalanced on the individual level. The number of participants was determined to be within the range of related brain stimulation studies [17,22,37].

The experimental sessions were scheduled at least 4 days apart from each other. Each session lasted on average 75 min in total, but the task and the subsequent fairness and social appropriateness ratings took a maximum of 35 min, which is within the time window for behavioural effects of the applied TMS protocol [38]. Participants received a participation fee of €7.50 for each session. Decisions in the task were incentivized and participants could earn extra money (€0 to €25.30) in each session depending on their decisions and the decisions of the individuals they were matched with. Social appropriateness ratings also were incentivized [33], using a coordination game to elicit a descriptive social norm. On average, participants earned €25.80 (s.d. = €6.60) per experimental session.

#### (i) Transcranial magnetic stimulation procedures

We used a double-cone TMS coil given its balance between depth of penetration and focality. Its geometry makes it is suitable for reaching deep regions in the brain (i.e. the mPFC in our study), while still producing a focal stimulation [39]. A continuous theta-burst stimulation protocol was employed (for details see electronic supplementary material), as it has been shown to produce consistent and rapid electrophysiological and behavioural changes that last for approximately 60 min after stimulation, which is considerably longer than for other stimulation protocols [38]. The stimulation sites were localized using the international 10–20 EEG system. While this approach may lead to inter-individual variability in which specific subparts of a brain structure are stimulated, this should not be problematic in our study considering that the target cortical areas are large and we used a double-cone coil, which is less focal than other coils [40].

To stimulate the rDLPFC, we placed the centre of the TMS coil on the electrode site F4 while holding the handle of the coil parallel to the cortical midline. The mPFC was stimulated by placing the coil between Fp1 and Fp2 while holding the handle of the coil at a 45 degrees angle from the cortical midline. The potential cortical reach of the stimulation on the target brain areas with the double-cone TMS coil was modelled on a template brain with SimNIBS [41]. Results of the simulation show that by placing the coil on the electrode sites F4 and between Fp1 and Fp2, the strength of the electric field would be the highest over the rDLPFC and mPFC, respectively (figure 1*b*).

For sham stimulation, the same protocol was used; however, this time the coil was tilted by 180 degrees while keeping coil orientation and intensity the same as in the TMS conditions. This ensured that participants had the same auditory stimulation, but did not receive magnetic stimulation of the brain. In the sham condition, the coil was placed on the middle point between the stimulation sites of the rDLPFC and the mPFC.

### (b) Experimental task: dictator game with punishment and reward

In each session, participants made decisions in 140 trials. In each trial, three individuals were randomly matched and assigned three different roles, Proposer, Receiver and Observer in a dictator game with the possibility of punishment and reward. Participants always made decisions in the role of Receiver or Observer (within-subject). Proposer behaviour was collected in a separate experiment (see electronic supplementary material). In each trial, the Proposer was endowed with 240 experimental currency units (ECUs), the Receiver with zero ECUs and the Observer with 200 ECUs. All participants were informed that at the end of the experiment earned ECUs would be converted to euros at a rate of €0.10/ECU.

In each trial, a different Proposer decided on how to allocate their endowment between themselves and the Receiver. The proposed allocation was seen by the participant irrespective of their role (Receiver, Observer). Proposers could choose between different allocation bundles: 200–40 (Very Greedy), 160–80 (Greedy), 120–120 (Equal), 80–160 (Generous) and 40–200 (Very Generous), where the first term refers to what the Proposer kept for themselves and the second term to what was allocated to the Receiver. For each of these bundles, receivers could get any allocation within the range of ±3 ECUs from the original choice (electronic supplementary material, table S5). The terms ‘Very Greedy’, etc. were not used in the experiment.

After learning the allocation decision made by the Proposer, participants could punish or reward the Proposer by decreasing or increasing the Proposer's payoff. A 3 × 2 × 2 within-subjects experiment design was implemented in which, besides the already described three TMS sessions, both personal involvement (Receiver and Observer, respectively) and the cost to punish or reward the Proposer (Cost and No Cost, respectively) varied.

In the Receiver No Cost (Rec_NC) and the Observer No Cost (Obs_NC) conditions, punishing or rewarding the Proposer was not costly: every ECU invested by the Receiver (Observer) in punishment or reward reduced or increased the Proposers' payoff by 5 ECUs but did not affect the Receiver's (Observer's) payoff. In the Receiver with Cost (Rec_C) and the Observer with Cost (Obs_C) conditions, each ECU invested in rewarding (punishing) the Proposer increased (reduced) the Proposer's payoff by 5 ECU and reduced the Receiver's (Observer's) payoff by 1 ECU.

In all four conditions (Rec_C, Rec_NC, Obs_C and Obs_NC), participants were informed that they could invest up to 40 ECUs to reward or punish the Proposer. This ensured that irrespective of the Proposer's allocation decision, the Receiver and Observer, respectively, always would have enough ECUs to invest in punishing or rewarding the Proposer. Moreover, participants were informed that they could not decrease Proposers' payoff below 0 or increase Proposers’ payoff above 240 ECUs. These design features ensured that participants could never incur losses themselves nor cause losses to Proposers.

In each condition, the participant and the Proposer were the only individuals who knew the punishment or reward decision taken by the participant. Thus, in Rec_C and Rec_NC, a participant's decision as Receiver could not be seen by the individual who was in the role of Observer. Likewise, in Obs_C and Obs_NC, a participant's decision as Observer could not be seen by the individual in the role of Receiver. This ensured that the decisions to punish and reward made by participants could not be influenced by reputation or image concerns that might ensue from being observed. Moreover, the design ensured that in each condition there was only one person who could modify the Proposer's payoff and one person who could only passively watch the behaviour of the Proposer. Hence, information was symmetric across all conditions.

In each of the three sessions, participants faced the four different conditions in a randomized block design. In each block, participants went through 35 trials in a given condition, each presenting a different allocation by different Proposers (presented in random order and varying over the full range of Very Greedy to Very Generous allocations). For each allocation, participants decided how much to reward or punish the Proposer. After a block for a given condition ended, a new block started featuring a new condition. During each session, facial expressions of participants were recorded with a webcam. Results of facial expression analysis are not reported here.

At the end of each session, one trial was selected randomly for payment and all three individuals in the different roles in that trial were paid according to the decisions made in that trial. Earnings in a session and total earnings were revealed to participants only at the end of the last session to avoid session earnings effects on decisions in subsequent sessions. All earnings, including participation fees, were transferred to participants' bank accounts within one week of their last session (electronic supplementary material, table S4).

### (c) Measures of fairness and social appropriateness of Proposer allocations

Immediately after the trials involving the Dictator game with Punishment and Reward, participants completed two tasks to test whether brain stimulation affected the perceptions of what constitutes fair and socially appropriate allocations, respectively. Fairness ratings measure the perceived fairness of allocations and social appropriateness judgements elicit participants’ descriptive social norms regarding allocations. Fairness and appropriateness ratings were presented in a counterbalanced order and completed within the range of effectiveness of the TMS intervention. For the fairness ratings, participants were asked to assess, for each of the four conditions, the fairness of each bundle allocation available to Proposers on a 7-point Likert-scale ranging from ‘Very unfair’ to ‘Very fair’.

For the appropriateness ratings, participants were asked to rate the social appropriateness of the five possible Proposer's bundle allocations in each condition using an incentive-compatible norm elicitation task [33]. Social appropriateness was described to participants as the ‘behaviour that most people agree is the correct or ethical thing to do’. Appropriateness was rated on a 4-point Likert-scale that ranged from ‘Very socially inappropriate’ to ‘Very socially appropriate’. At the end of each session, one of the Proposer's allocations was randomly selected. Participants' appropriateness rating for the selected allocation was then compared to the most frequent answer for the same allocation provided by another group of individuals, whose answers were collected in a separate online experiment that took place before the first TMS session. Collecting answers from a separate group of participants, which were not exposed to brain stimulation, allows avoiding both deception and any bias due to TMS. Individuals participating in this online experiment received information about the four different conditions and were asked to rate, for each condition, the social appropriateness of each of the five allocation bundles that the Proposer could choose. Their ratings were incentivized with a €5 payment if their appropriateness judgement matched the most frequent answer of all online participants; €0 otherwise. Each participant in the main experiment received €5 if their social appropriateness judgement matched the most frequent answer in the separate online experiment; €0 otherwise. Importantly, participants have an incentive to reveal what they perceive to be the collectively shared judgement of appropriateness of the allocations they evaluate (i.e. descriptive social norm), and not their own personal norm [33]. Participants were informed about the incentives before they made their rating decision but learned their earnings from the appropriateness task only at the end of the last experimental session.

## 4. Results

Counterintuitive rewarding of (Very) Greedy allocations was extremely rare (less than 5.2% of all possible cases), as was counterintuitive punishment of Equal and (Very) Generous allocations (less than 6.4%). Therefore, when analysing punishment, we focused on trials where participants faced Very Greedy and Greedy allocations, and when analysing reward, we used Equal, Generous and Very Generous allocations. Equal allocations were included in the reward domain because of evidence showing that the equal split of the endowment in dictator games is considered the most socially appropriate outcome [18,33]. Our hypotheses predict a decrease in punishment and reward after TMS relative to sham. Thus, to be able to detect this reduction, it is important to observe frequent individual punishment and reward in the sham session. To avoid a floor effect and to minimize the likelihood of false negatives we excluded eight participants who, in the sham condition, punished and rewarded Proposers in less than 50% of all trials across the four experimental conditions (final sample: *N* = 25, mean age = 22.28, s.d. = 3.64, 19 women). Importantly, even if these Weak Reciprocators are included in the analysis only a few differences are observed, which we report in the discussion section.

In our analysis, we exploit the within-subjects nature of our design and control for baseline effects by employing panel data random-effects Tobit (reTobit) regressions left-censored at zero (no punishment/reward) and right-censored at 200 (full punishment/reward) with s.e. clustered at the participant level. The models also include random intercepts for subjects to account for cross-subject variability as well as controls for gender, fairness perception, social appropriateness ratings, trial order within an experimental session and session number. Full regression tables are reported in the electronic supplementary material, tables S1, S8 and S11. Guided by our preregistered hypotheses, we conducted post-estimation two-sided marginal effects tests to compare behaviour in each experimental condition under brain stimulation and sham (electronic supplementary material, tables S2, S3, S9, S10, S12 and S13). Reported *p*-values survive multiple comparisons correction using the false discovery rate (FDR) [42] at the 95% confidence level, unless otherwise stated (for details see electronic supplementary material).

### (a) Evidence for reciprocal punishment and reward

Before reporting the effect of TMS, we use the sham condition to examine whether participants engage in reciprocal fairness at all. Consistent with existing evidence [6,7,10,19], we find that average punishment of (Very) Greedy allocations by both Receivers and Observers is significantly different from zero when punishment is non-costly and when it is costly (*p* < 0.001, reTobit Wald test). Similarly, average reward of Equal and (Very) Generous allocations is significantly different from zero irrespective of participants’ role and cost condition (*p* < 0.001, reTobit Wald test). Thus, participants act reciprocally fair in all conditions, albeit both punishment and reward are smaller when costly than when non-costly (*p* *<* 0.001, reTobit) (for a more detailed discussion, see electronic supplementary material, figure S1 and table S6).

### (b) Transcranial magnetic stimulation over the right dorsolateral and medial prefrontal cortex reduces reciprocal punishment when costly

**Figure 2.**
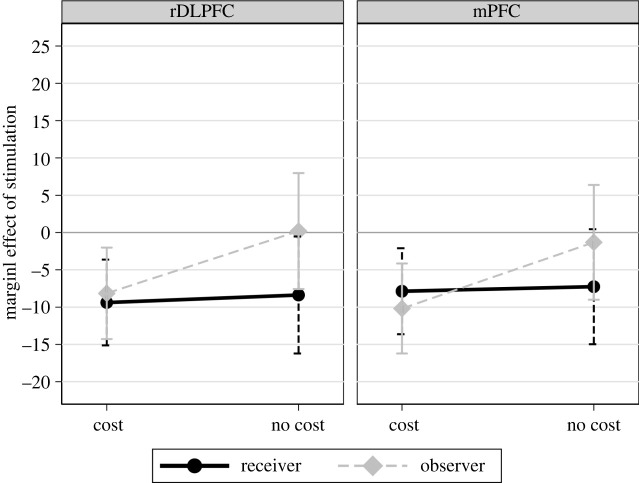
Effect of TMS on punishment. Marginal effect of rDLPFC and mPFC stimulation versus sham on punishment of (Very) Greedy allocations. The horizontal solid line at zero indicates the point estimate for the sham stimulation relative to the stimulation of rDLPFC and mPFC, respectively, in the different conditions. The black circle and the grey diamond markers represent marginal effects of TMS compared to sham for Receiver and Observer roles. Bars indicate the 95% confidence intervals based on regression analysis. (Online version in colour.)

Brain stimulation of the rDLPFC and the mPFC strongly affects reciprocal punishment of (Very) Greedy allocations (figure 2; electronic supplementary material, tables S1 and S2). Estimated marginal means derived from the three-way interaction between rDLPFC stimulation, cost and personal involvement reveal that, compared to sham, TMS over the rDLPFC decreases both costly and non-costly punishment when participants are in the Receiver role (Rec_C: *t* = −9.38, *p* = 0.001, d.f. = 1, 95%CI [−15.13, −3.63]; Rec_NC: *t* = −8.37, *p* = 0.036, d.f. = 1, 95%CI [−16.21, −0.53]; reTobit), and costly punishment when participants are in the Observer role (Obs_C: *t* = −8.15, *p* = 0.009, d.f. = 1, 95%CI [−14.28, −2.02]). When punishment is non-costly TMS over the rDLPFC has no effect for Observers (Obs_NC: *t* = 0.19, *p* *=* 0.96, d.f. = 1, 95%CI [−7.58, 7.96]). Results are similar following TMS over the mPFC. Relative to sham, costly punishment decreases for both Receiver and Observer roles (Rec_C *t* = −7.87, *p* = 0.007, d.f. = 1, 95%CI [−13.63, −2.11]; Obs_C: *t* = –10.18, *p* = 0.001, d.f. = 1, 95%CI [−16.21, −4.15]). When punishment is non-costly, a tendency to decrease it is observed for the Receiver role (Rec_NC: *t* = −7.26, *p* = 0.065, FDR corrected < 0.10, d.f. = 1, 95%CI [−14.97, 0.44]), but not for the Observer role (Obs_NC: *t* = −1.31, *p* *=* 0.74, d.f. = 1, 95%CI [−8.99, 6.37]).

The result that brain stimulation of the rDLPFC decreases costly punishment is consistent with the inhibitory *cognitive control* hypothesis. Previous studies have reported this for second-party interaction [15,16]. Here, we show that it also applies in the third-party context. However, this hypothesis is only partially supported as it does not predict the observed reduction of punishment in the non-costly receiver condition. As TMS over the rDLPFC does not reduce punishment in all conditions the *domain-general* hypothesis is not supported. The result that TMS over the mPFC decreases costly punishment for second-party but not for non-costly third-party interactions is consistent with previous studies [21,22]. However, the *self-referential* hypothesis suggested by these studies is not fully supported because TMS over the mPFC also reduces punishment by Observers when it is costly. This suggests that the mPFC is involved in *cognitive control* of selfish impulses.

### (c) Transcranial magnetic stimulation over the right dorsolateral and medial prefrontal cortex tends to decrease reciprocal reward when costly but increases it when non-costly

**Figure 3.**
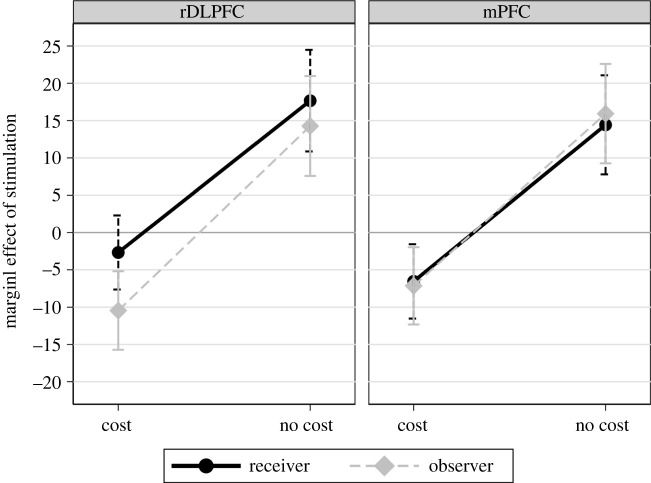
Effect of TMS on reward. Marginal effect of rDLPFC and mPFC stimulation versus sham on reward of Equal and (Very) Generous allocations. The horizontal solid line at zero indicates the point estimate for sham stimulation relative to the stimulation of rDLPFC and mPFC, respectively, in the different conditions. The black circle and the grey diamond markers represent marginal effects of TMS compared to sham for Receiver and Observer roles. Bars indicate the 95% confidence intervals based on regression analysis. (Online version in colour.)

Results for the effect of TMS over the rDLPFC and the mPFC on reciprocal reward in response to Equal and (Very) Generous allocations are remarkably different from the effect on punishment (figure 3; electronic supplementary material, tables S1 and S3). Estimated marginal means derived from the three-way interaction between rDLPFC stimulation, cost and personal involvement show that, compared to sham, TMS over the rDLPFC decreases costly reward when participants are in the Observer role (Obs_C: *t* = –10.46, *p* < 0.001, d.f. = 1, 95%CI [−15.72, −5.2]), but not when in the Receiver role (Rec_C: *t *= –2.68, *p* *=* 0.29, d.f. = 1, 95%CI [−7.65, 2.29]). By stark contrast, and contrary to any of the proposed mechanisms, TMS over the rDLPFC strongly increases non-costly reward relative to sham, for both Receiver and Observer roles (Rec_NC: *t* = 17.67, *p* < 0.001, d.f. = 1, 95%CI [10.86, 24.48]; Obs_NC: *t* = 14.27, *p* < 0.001, d.f. = 1, 95%CI [7.58, 20.97]). Results following TMS over the mPFC are only slightly different. When costly, reciprocal reward decreases for both Receiver and Observer roles (Rec_C: *t* = −6.55, *p* = 0.01, d.f. = 1, 95%CI [−11.53, −1.57]; Obs_C: *t* = −7.14, *p* = 0.007, d.f. = 1, 95%CI [−12.32, −1.97]), but strongly increases when rewards are non-costly (Rec_NC: *t* = 14.43, *p* < 0.001, d.f. = 1, 95%CI [7.79, 21.07]; Obs_NC: *t* = 15.93, *p* < 0.001, d.f. = 1, 95%CI [9.27, 22.6]).

The decrease in costly reward for Observers, but not for Receivers following TMS over the rDLPFC, is inconsistent with the *cognitive control* hypothesis. Moreover, as we do not find a decrease in reward across all experimental conditions, the *domain-general* hypothesis is not supported for the rDLPFC in the reward domain. For the mPFC, our findings are consistent with the *cognitive control* hypothesis, as stimulation of this brain area leads to a decrease of costly reward irrespective of personal involvement. As TMS over the mPFC decreases costly reward also when being an Observer, we do not find support for the *self-referential* mechanism in the reward domain. The increase of non-costly reward after TMS over the rDLPFC and the mPFC relative to sham has not been predicted by any of the hypotheses. We discuss this in more detail below.

### (d) Brain stimulation does not affect perceived fairness and social appropriateness

**Figure 4.**
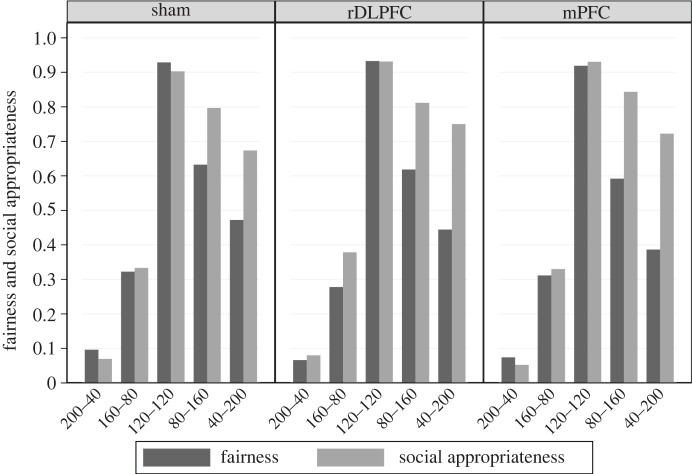
Fairness and appropriateness ratings across allocations by brain stimulation condition. Ratings are normalized to vary between 0 (Very unfair/socially inappropriate) and 1 (Very fair/socially appropriate).

In all brain stimulation conditions, the equal split is rated as the fairest and most socially appropriate allocation, whereas (Very) Greedy allocations are rated as (very) unfair and socially inappropriate. Interestingly, (Very) Generous allocations are perceived as less fair and rated as less socially appropriate than the equal split (figure 4). Neither perceived fairness nor social appropriateness ratings differ across TMS conditions (Friedman test, Fairness *p* = 0.556; Appropriateness *p* = 0.418) and, thus, brain stimulation of the rDLPFC or the mPFC does not alter them, as hypothesized. The pattern of perceived fairness and social appropriateness ratings is similar across player roles and cost conditions of punishment and reward (electronic supplementary material, figure S2). Thus, the observed effects of TMS over the rDLPFC and the mPFC on reciprocal fairness cannot be attributed to a change in perceived fairness or the social appropriateness of allocations.

## 5. Discussion

We tested hypotheses related to three cognitive mechanisms derived from existing evidence on reciprocal fairness (*cognitive control, domain-general* and *self-reference)* and find that none of them can fully account for all results. The *cognitive control* and the *self-referential* mechanisms are only partially supported, as we also find effects that are not predicted by these mechanisms. In addition, we do not find evidence consistent with the *domain-general* mechanism. We note, however, that a broader interpretation of the *domain-general* mechanism may refer to a value computation system that is sensitive to the overall number of variables to be considered. Arguably, when reciprocal fairness is costly, more information needs to be integrated and computed than when it is not costly. Based on this interpretation, brain stimulation of the rDLPFC should primarily disrupt reciprocal fairness in the costly conditions, a prediction that is only partially borne out by the data. Importantly, none of the hypothesized mechanisms predicted the asymmetric response in the punishment and reward domain, specifically the increase in non-costly reward after TMS over either brain area was unexpected. In the following, we propose mechanisms reconciling these findings.

Comparisons of estimated marginal means in the punishment domain reveal that TMS over the rDLPFC and mPFC decreased punishment in comparison to sham in all conditions, except the non-costly Observer condition, where any component of self-relevance (i.e. economic cost and being target) is absent. This is the only condition where the participant is not the target of unfairness and bears no costs of punishment. Thus, our results point to the possibility that the rDLPFC and the mPFC respond to both the personal material costs of punishment and personal involvement. In our view, this implies the need of revisiting the *cognitive control,* and *self-reference* mechanisms in an attempt to unify these two accounts into a more broadly defined self-relevance mechanism, which includes not only the personal component of being the target of someone's action, but also the material component of self-interest. Such a broader perspective of self-relevance is consistent with evidence showing that lateral prefrontal brain regions, in addition to cortical midline structures, are engaged in self-referential tasks with a strong cognitive component [31,32]. It is also in line with previous studies reporting the involvement of the mPFC in cognitive control [43] and resource maximization [44]. We posit that both the rDLPFC and mPFC may be sensitive to contextual cues of self-relevance and contribute to the generation of an appropriate reciprocal response in a context-dependent manner. That is, by controlling economic selfishness when costs are present or enabling self-referential processes when one is the target of unfairness. Thus, when TMS is applied to these regions, the willingness to (costly) enforce fairness may be diminished by a combination of both increased economic selfishness and decreased self-reference. Future studies should scrutinize this interpretation.

The robust increase of non-costly reciprocal reward following TMS over the rDLPFC or the mPFC was unanticipated. Although disruption of the prefrontal cortex has been previously associated with an increase in costly sharing [45], our results are unlikely to be due to a general increase in generosity, because in our study, the observed increase in reward is restricted to conditions where reciprocal fairness is non-costly. Our interpretation of this unanticipated intriguing result relates to the three distinct motivational building blocks of reciprocal reward: material selfishness, fairness-norm enforcement, and reciprocity. We start with some observations regarding these building blocks. First, the selfishness motive prescribes that an individual should never punish or reward others when it is costly to do so but may be indifferent when it is non-costly. Second, fairness-norm enforcement predicts that the more (less) fair and socially appropriate an allocation is the more it should be rewarded (punished). Third, reciprocity comprises acting (un)kindly in response to a (un)kind act irrespective of the fairness norm; that is, compared to the Equal allocation, (Very) Greedy allocations should be punished more, because they are unkinder, and (Very) generous allocations should be rewarded more, because they are kinder. In a separate survey, we confirm that participants indeed rate (Very) Generous allocations significantly kinder and (Very) Greedy allocations significantly unkinder than Equal allocations (Wilcoxon signed-rank test, *p* < 0.001) (electronic supplementary material, figure S5).

Applying these considerations, it follows that in the punishment domain, both fairness-norm enforcement and pure reciprocity lead to the same behavioural predictions: (Very) Greedy allocations are punished because they deviate from the fairest allocation and because they are unkind. By contrast, in the reward domain, these two motives steer behaviour into different directions because allocations that are kinder than the equal split are perceived as less fair and less socially appropriate (figure 4). Therefore, for (Very) Generous allocations, fairness-norm enforcement predicts less reciprocal reward whereas reciprocity predicts more reciprocal reward. Indeed, under sham (Very) Generous allocations are rewarded more than the equal split (*p* < 0.001, reTobit), providing evidence for the existence of a reciprocity motive (electronic supplementary material, table S7).

Consequently, if TMS over the rDLPFC and the mPFC suppresses fairness-norm enforcement but not the reciprocity motive, then the non-costly reward of (Very) Generous allocations should increase. An Equal allocation can be rewarded out of fairness-norm enforcement and reciprocity motives, implying that rewarding of Equal allocations may or may not decrease after TMS over the rDLPFC and the mPFC relative to sham, depending on the relative contribution of these motives. Further analysis provides support for these predictions. It shows that TMS over either region increases non-costly reward of (Very) Generous allocations and the Equal allocation (reTobit, *p* < 0.001; electronic supplementary material, tables S8–S10 and figures S3 and S4). Taken together, the result of increased non-costly reward of Equal and (Very) Generous allocations after stimulation of the target brain areas is consistent with the interpretation that these brain areas are involved in both implementing the fairness-norm enforcement motive and controlling the reciprocity motive.

When reward is costly, fairness-norm enforcement and pure reciprocity need to be traded-off with the selfish motive of cost-saving, which decreases reciprocal fairness. Consistent with this we find that in sham reciprocal reward is substantially weaker with than without costs (electronic supplementary material, figure S1). When TMS is applied to the rDLPFC and the mPFC, we observe a general decrease of costly reciprocal reward. This indicates that when reward is costly the target brain regions are also involved in controlling selfish behaviour.

After TMS is applied to either the rDLPFC or the mPFC, we observe a similarity in the behavioural pattern. One possible explanation for this could be that the stimulation of brain regions was not focal enough. We consider this unlikely because, first, the two investigated regions are sufficiently distant from each other so that TMS effects should be specific, and second, the coil used is known for its good balance between depth and focality [39]. Indeed, a SimNIBS TMS simulation [41] shows that although TMS expands beyond the target areas, the strength of the electric field in the mPFC when stimulating the rDLPFC (and vice versa) is very low (figure 1*b*). However, it cannot be ruled out that the stimulation of one region may have affected the functioning of the other region through a network effect. Reciprocal fairness is a complex behaviour and it is unlikely that its implementation is restricted to a single area of the frontal cortex [17]. In addition, because the rDLPFC and the mPFC are involved in many cognitive processes (e.g. value-based decision-making [46], theory of mind [47,48]), brain stimulation over these regions could have simultaneously impacted different mental abilities. This may have reduced the ability to observe effects restricted to only our hypothesized cognitive processes. Future studies could focus on unravelling the functional specificities of the investigated regions as well as network effects related to reciprocal fairness. The combination of functional magnetic resonance imaging and TMS may be of great value to this endeavour.

As noted, in the main analysis, we excluded eight Weak Reciprocators to avoid floor effects and false negatives. Most results are robust to including these outliers. When including those weak reciprocators, we see somewhat weaker TMS effects in some conditions of costly reward and punishment. As reciprocal fairness is generally weaker in costly than in non-costly situations, this is consistent with a floor effect. Importantly, the reported main results hold for a large majority of our participants (see electronic supplementary material, figures S6 and S7 and tables S11–S14).

Our analytical sample size is in keeping with previously published literature on the neural mechanisms of reciprocal fairness [15,17,20,22] and with other brain stimulation studies employing within-subjects designs [16,18,47]. We also made efforts to increase power by employing a within-subjects design with a large number of trials per condition which is known to increase power [49]. Still, we acknowledge that the sample size could be a limiting factor regarding the robustness of our results. Future research should attempt to replicate and extend our findings.

Our hypotheses were formulated assuming that the habitual response of the average individual would be economically selfish and that cognitive control would be needed to overwrite it. Our data suggest that, on average, this was indeed the case in our sample, considering that punishment and reward in the sham condition were less frequent when reciprocity was costly. Moreover, both the observed decrease in costly punishment and costly reward after TMS speak in favour of economic selfishness being the average habitual response. If individuals' habitual response would be to reciprocate (un)fairness, cognitive control would be needed to regulate such reciprocal response to avoid that individuals would hurt their economic interests too much. In this case, TMS would be expected to increase both punishment and reward, especially in the costly conditions. Yet, we only observe such a pattern for non-costly reward. Nevertheless, we acknowledge that habitual responses can vary across individuals, and that the role cognitive control plays in reciprocal fairness may differ depending on whether individuals’ intuition is to behave in a selfish or reciprocal fair manner. Thus, our results should be interpreted as applying to our study population, on average. Exploring individual differences regarding habitual responses to (un)fairness and the role of the investigated brain areas in it is a promising line of future research.

By directly comparing reciprocal punishment and reward within-subjects, we provide evidence of behavioural and neural asymmetries between these two domains, consistent with the scarce behavioural evidence suggesting asymmetries in reciprocation to kind and unkind behaviour [50,51]. To reconcile these asymmetries, we propose a novel interpretation of the functional roles of the rDLPFC and the mPFC in reciprocal fairness. In reciprocal punishment, the rDLPFC and the mPFC process self-relevant information, both in terms of personal cost and personal involvement. For reciprocal reward, the rDLPFC and the mPFC are involved in controlling both selfish and reciprocity motives, while simultaneously promoting the enforcement of fairness norms.

In our analyses we specifically investigate reciprocal punishment of Very Greedy and Greedy offers and reciprocal reward of Equal, Generous and Very Generous offers. Further exploratory analyses considering all trials, including counterintuitive reward of Very Greedy and Greedy offers and counterintuitive punishment of Equal, Generous and Very Generous offers, respectively, suggest that neural modulation of reciprocal fairness is inequity-dependent for the rDLPFC. That is, TMS effects on reciprocal punishment are only observed for Very Greedy and Greedy offers, whereas TMS effects on reciprocal reward are solely present for Equal, Generous and Very Generous offers. Regarding the mPFC, we observe that the effect of TMS seems to be more inequity-independent: TMS over mPFC reduces also punishment of Equal, Generous and Very Generous offers and tends to increase reward of Very Greedy and Greedy offers compared to sham (see electronic supplementary material, table S15). Since counterintuitive punishment and reward were quite rare in our sample (less than 5% of cases), we note that these results need to be interpreted with caution. Nonetheless, they indicate a potential interesting asymmetry between the involvement of the rDLPFC and mPFC in reciprocal fairness, which may merit future investigation.

To conclude, we show that both the rDLPFC and the mPFC are implicated in reciprocal punishment as well as in reciprocal reward, but that their involvement in these domains is not symmetric. This finding indicates that reciprocal punishment and reward are not simply two sides of the same coin, which is relevant for the research program of building neurobiologically plausible models of social decision-making [52]. This will help to achieve better results in the intricate task of predicting human social behaviour. Our findings can also be of relevance for applied settings where brain stimulation may be used for interventions to induce behavioural change. Although our study only investigated transient effects, we believe that our finding that brain stimulation can increase reciprocal reward may open interesting avenues for neurological interventions to increase pro-sociality.

## Data Availability

Data and code used for this study can be found at the following link: https://osf.io/ab89m [53]. Additional information is provided in the electronic supplementary material [54].
